# Role of microRNA in the risk stratification of ischemic strokes

**DOI:** 10.3389/fneur.2025.1499493

**Published:** 2025-02-12

**Authors:** Hosam M. Al-Jehani, Ahmed Hafez Mousa, May Adel Alhamid, Fawaz Al-Mufti

**Affiliations:** ^1^Department of Neurosurgery and Interventional Neuroradiology and Critical Care Medicine, Imam Abdulrahman Bin Faisal University, Al Khobar, Saudi Arabia; ^2^Department of Neurology and Neurosurgery, Montreal Neurological Institute and Hospital, McGill University, Montreal, QC, Canada; ^3^Department of Neurosurgery, Houston Methodist Hospital, Weill Cornell University, Houston, TX, United States; ^4^Department of Neurosurgery and Interventional Neuroradiology, King Fahd Hospital Specialist Hospital, Dammam, Saudi Arabia; ^5^Neurosciences, Dubai Health, Dubai, United Arab Emirates; ^6^Department of Neurosurgery, Graduate Medical Education, Mohammed Bin Rashid University of Medicine and Health Sciences, Dubai, United Arab Emirates; ^7^Department of Neurosurgery and Interventional Neuroradiology, Imam Abdulrahman Bin Faisal University, Al Khobar, Saudi Arabia; ^8^Department of Neurology and Interventional Neuroradiology, King Fahad Hospital of the University, Imam Abdulrahman Bin Faisal University, Dammam, Saudi Arabia; ^9^Department of Neurology and Neurosurgery, Westchester Medical Center at New York Medical College, Valhalla, NY, United States

**Keywords:** ischemic stroke, microRNAs, inflammatory response, neuronal death, vascular inflammation, endothelial dysfunction, thrombus formation

## Abstract

**Background:**

Ischemic stroke is a major cause of death and morbidity, and risk classification is essential for predicting therapeutic outcomes. MicroRNAs may be useful indicators for risk stratification, as they control gene expression and influence physiological and pathological processes.

**Methodology:**

A systematic strategy was developed to search relevant material using databases like PubMed, Scopus, and Web of Science. Selection criteria included human research, a certain date, or categories of studies. Data extraction, synthesis, and analysis were carried out to find trends, similarities, and differences among the chosen studies. The study’s design, sample size, methodology, statistical analysis, and any potential biases or restrictions from the selected reference papers were also taken into account.

**Results and findings:**

MicroRNA is an important biomarker for risk stratification in Ischemic Strokes. It can be used to identify Stroke-Specific microRNA Signatures, identify diagnostic and prognostic values, and regulate Vascular Inflammation, Endothelial Dysfunction, and Thrombus Formation and Resolution. It also has potential therapeutic applications.

**Conclusion:**

MicroRNAs have emerged as promising biomarkers for predicting stroke risk, severity of strokes, and clinical outcomes. They can be used to predict the severity of a stroke and aid clinicians in making treatment decisions.

## Introduction

Ischemic stroke are frequent cerebrovascular events defined as an abrupt reduction in blood flow to the brain, which causes tissue damage and neurological impairments. It is a major contributor to death and morbidity on a global scale. For patients with ischemic stroke, effective risk classification is essential for predicting outcomes and directing therapy choices. A number of variables, including age, sex, medical history, and imaging results, are generally considered to determine further risk classification, have been studied extensively. Most of the work up done is after the event happens and fails to identify the individual’s specific risk of stroke occurrence or recurrence. MicroRNAs (miRNAs) may be useful indicators for ischemic stroke risk stratification, according to recently published research papers (1, 2).

MicroRNAs are tiny, non-coding RNA molecules that control gene expression by attaching to and degrading or inhibiting the translation of their target messenger RNAs (mRNAs). Numerous physiological and pathological processes, such as neuronal growth, synaptic plasticity, and neuroinflammation, are influenced by miRNAs. Dysregulation of some miRNAs has been connected to the etiology of ischemic stroke and may provide important clues for stroke risk assessment (3–5).

Numerous studies have shown that ischemic stroke patients’ miRNA expression patterns are different from those of healthy controls. The molecular processes implicated in the pathogenesis of stroke, including as inflammation, oxidative stress, apoptosis, angiogenesis, and neuroplasticity, are influenced by these differentially expressed miRNAs. Notably, several miRNAs have consistently displayed dysregulation in many investigations, suggesting their potential as accurate biomarkers for predicting the risk of stroke (6, 7).

In a variety of biofluids, such as blood, cerebrospinal fluid, and saliva, miRNAs may be found and measured. They make good candidates for clinical applications because of their non-invasive nature. The discovery of certain miRNA signatures linked to various stroke subtypes, severity, and prognosis has also been made easier because to developments in high-throughput technologies like microarray analysis and next-generation sequencing (8).

It is very promising to employ miRNAs as biomarkers for ischemic stroke risk stratification. MiRNA profiling may improve the accuracy of current risk prediction methods and enable individualized treatment approaches. Additionally, miRNAs may be used as therapeutic targets for stroke since altering their expression may be able to affect pathways linked to stroke and aid in neuroprotection and recovery (9).

In nutshell research into the role of miRNAs in the risk assessment of ischemic strokes is still in its infancy. MiRNAs have distinctive patterns of expression in stroke patients and are engaged in a number of molecular processes that contribute to the pathophysiology of stroke. MiRNA profiles may enhance the predictive accuracy of current risk prediction algorithms and help to individualized stroke therapy. To confirm the clinical usefulness of miRNAs and investigate their potential therapeutic uses in ischemic stroke, more research is necessary. It could be able to create risk stratification models for ischemic strokes that are more precise by combining miRNA profiling with conventional risk factors including age, sex, hypertension, diabetes, and smoking history. In turn, this can aid in locating stroke-at-risk people who may benefit from proactive measures and specialized treatment plans to lower their risk. Present review article focuses on the role of microRNA in the risk stratification of Ischemic Strokes. MicroRNAs (miRNAs) are promising indicators for ischemic stroke risk stratification due to their stability in biofluids, dynamic expression changes in response to stroke-related pathophysiological processes, and role in regulating gene expression. Unlike DNA or mRNA, miRNAs provide a more sensitive and minimally invasive means to assess stroke risk and progression. Their ability to reflect inflammation, endothelial dysfunction, and neuronal injury makes them uniquely relevant for personalized risk assessment and monitoring.

## Objectives

- To review the current knowledge on the association between microRNAs and ischemic strokes.
- To identify the gaps in knowledge and highlight the areas that require further research to establish the utility of microRNAs as risk stratification biomarkers in ischemic strokes.

## Methodology

- Research question: Role of microRNA in the risk stratification of ischemic strokes?
- Study Design: Narrative Review.
- Search strategy: A review of the relevant literature was framed by identifying appropriate databases such as PubMed, Scopus, and Web of Science using relevant keywords, including “microRNA,” “risk stratification,” and “ischemic stroke.” Variations of these keywords and synonyms were also considered. Additionally, the reference lists of relevant articles was reviewed to identify additional sources.
- Selection criteria: Proper criteria for selecting relevant studies were established. These criteria may include the inclusion of human studies, a specific timeframe, or specific types of studies (e.g., clinical trials, observational studies). It was also ensured that the studies selected are directly related to the role of microRNA in risk stratification of ischemic strokes.
- Data extraction: Relevant data from the selected studies was extracted. This may include study characteristics (e.g., study design, sample size), microRNA-related information (e.g., microRNA types, expression levels), stroke-related information (e.g., stroke subtype, risk factors), and any other relevant findings or outcomes.
- Data synthesis and analysis: the extracted data was organized into meaningful categories or themes by identifying patterns, similarities, and differences across the selected studies. The findings in the context of the research question were analyzed looking for consistencies or discrepancies in the role of microRNA in risk stratification of ischemic strokes.
- Critical evaluation: the quality and reliability of the selected studies was assessed considering the study design, sample size, methodology, statistical analysis, and any potential biases or limitations and discussing the strengths and weaknesses of the evidence presented in each study.

## Results and important findings

### Role of microRNA in ischemic strokes

#### Dysregulation of microRNA in ischemic strokes

Small non-coding RNA molecules called microRNAs (miRNAs) are essential for the post-transcriptional control of gene expression. They participate in several biological processes, such as cellular differentiation, disease etiology, and development. MiRNA dysregulation has been linked to a number of illnesses, including ischemic strokes (7, 8, 10).

Ischemic stroke happens when the blood flow to the brain is interrupted, which causes the brain cells to perish from a lack of oxygen and nutrition. MiRNA dysregulation in ischemic strokes can take place on several levels and affect the pathophysiology of the disease (Table 1).

**Table 1:** Levels of dysregulation of microRNA in ischemic stroke (7, 10).

Study number	Levels of dysregulation	Pathophysiology
1	Modulation of the inflammatory response	Ischemic stroke causes a sophisticated inflammatory response in the brain. Dysregulated miRNAs can affect the expression of pro- or anti-inflammatory genes, which can influence this response. By targeting anti-inflammatory molecules, miR-155, for instance, has been demonstrated to enhance neuroinflammation, whereas miR-146a functions as a negative regulator of the inflammatory response.
2	Neuronal cell death	After an ischemic stroke, miRNAs can affect whether neurons survive or die. MiRNAs that target anti-apoptotic or genes involved in cell survival pathways, such miR-21 and miR-29a, have been reported to be increased in ischemic stroke. These miRNAs may accelerate neuronal cell death.
3	Blood–brain barrier integrity	The blood–brain barrier (BBB) is crucial for preserving brain homeostasis and safeguarding it from potentially hazardous chemicals. During an ischemic stroke, miRNA dysregulation can impact the integrity of the BBB. For instance, it has been demonstrated that the tight junction protein essential in maintaining the BBB, miR-132, targets it, potentially increasing permeability.
4.	Neurogenesis and angiogenesis	An ischemic stroke sets off a series of processes, including angiogenesis and neurogenesis, aimed at tissue healing. By altering the expression of genes involved in vascular expansion and neural differentiation, miRNAs can control these processes. MiR-126 and miR-210, for instance, have been linked to angiogenesis, whilst miR-124 and miR-137 have been linked to neurogenesis.

Gaining knowledge of the dysregulation of miRNAs in ischemic strokes might help identify possible treatment targets as well as the underlying biological causes. It’s crucial to remember that the area of miRNA research is still developing, and more study is required to understand the intricate relationships and pinpoint individual miRNA candidates for use in ischemic stroke diagnosis and treatment.

#### Impact of microRNA on ischemic stroke pathophysiology

The involvement of miRNAs in ischemic stroke has been extensively studied, and their dysregulation has been implicated in several aspects of stroke pathophysiology (Table 2).

**Table 2:** Pathophysiology and role of miRNAs in ischemic stroke (16, 22, 23).

Study number	Pathophysiology	Role of miRNAs
1	Neuronal cell death	Neuronal cell death brought on by an ischemic stroke is regulated by miRNAs, which have been linked to controlling apoptosis, or planned cell death, in neurons. By targeting anti-apoptotic genes or fostering pro-apoptotic signaling pathways, certain miRNAs, including miR-21, miR-29a, miR-34a, and miR-210, have been found to be increased in ischemic stroke and contribute to neuronal apoptosis.
2	Inflammation and immune response	The development of an ischemic stroke is significantly influenced by the inflammatory response. Following a stroke, miRNAs have been linked to the control of inflammatory and immunological responses. By targeting important genes involved in immune cell activation and cytokine production, for instance, miR-155, miR-146a, and miR-223 are involved in controlling the inflammatory response.
3	Blood–brain barrier (BBB) disruption	The breakdown of the BBB, which aids in keeping the brain microenvironment in a state of homeostasis, advances the course of an ischemic stroke. Matrix metalloproteinases (MMPs), which break down the extracellular matrix, and endothelial cell tight junction proteins are the targets of miRNAs, which have been demonstrated to modulate the integrity of the BBB. The BBB is regulated during a stroke by miRNAs such miR-155, miR-126, and miR-132.
4	Angiogenesis and neurovascular remodeling	Angiogenesis, the development of new blood vessels, and neurovascular remodeling are brought on by ischemic stroke. By focusing on genes involved in endothelial cell proliferation, migration, and vessel stability, miRNAs have been shown to regulate angiogenesis. It is known that miR-210, miR-132, and miR-126 contribute to angiogenesis and neurovascular remodeling after stroke.
5	Neuroplasticity and recovery	Long-term functional losses from ischemic stroke are common, yet the brain is capable of reorganization and rehabilitation. Numerous neuroplasticity-related processes, such as synaptic plasticity, neuronal differentiation, and axonal regeneration, have been revealed to be regulated by miRNAs. Examples of miRNAs that are involved in controlling neuroplasticity during stroke recovery include miR-134, miR-124, and miR-9.

Discovering new therapeutic targets for stroke treatment may be possible by better understanding the precise functions of miRNAs in the pathogenesis of ischemic stroke. To develop miRNA-based therapy strategies for stroke patients, more studies are required to clarify the intricate regulatory networks involving miRNAs and their target genes in the setting of ischemic stroke.

### MicroRNA as biomarkers for risk stratification

#### Identification of stroke-specific microRNA signatures

Small non-coding RNA molecules called microRNAs (miRNAs) are essential for controlling the expression of genes. They have been recognized as possible stroke biomarkers among other disorders. To aid in the early detection, diagnosis, and risk stratification of stroke, researchers have been looking into the identification of miRNA signatures that are unique to stroke (11).

Studies have compared the miRNA expression patterns of stroke patients and healthy people to find miRNAs that are differently expressed and linked to stroke. MiRNA expression patterns in blood, cerebrospinal fluid, and brain tissue from stroke patients have been examined using high-throughput methods such microarray analysis and next-generation sequencing (12, 13).

Specific miRNA signatures that are connected to stroke have been found by comparing the miRNA profiles of stroke patients and healthy controls. Depending on the kind and severity of the stroke, these stroke-specific miRNA signatures may include upregulated or downregulated miRNAs, and their expression levels may change (9).

#### Diagnostic and prognostic value of microRNA in ischemic strokes

The most frequent form of stroke, an ischemic stroke, is brought on by a blockage in a blood artery feeding the brain. In ischemic strokes, microRNAs have demonstrated encouraging diagnostic and prognostic usefulness. Specific miRNAs that can identify ischemic stroke patients from healthy people or those who have had other types of strokes have been found in several studies (2, 14).

### Diagnostic value

It has been reported that a number of miRNAs, including miR-124, miR-125b, miR-133a, and miR-210, are increased in the blood or cerebrospinal fluid of people who have had ischemic strokes. These miRNAs may be used as possible diagnostic biomarkers to help identify and classify ischemic strokes earlier.

### Prognostic value

The severity of ischemic strokes and their clinical consequences have been linked to the expression levels of certain miRNAs. In individuals with ischemic stroke, increasing levels of miR-23a and miR-221 have been linked to poorer neurological outcomes and higher mortality. Decisions about therapy and prognosis may be aided by tracking the expression of these miRNAs.

#### Association of microRNA with stroke subtypes and severity

Stroke is a diverse disorder with several subgroups, including stroke of other determined or unexplained origin, cardio embolism, small vessel disease, and stroke of big artery atherosclerosis. MicroRNAs have been linked to various stroke subtypes and can shed light on the pathophysiology and underlying causes (2, 12, 15).

It has been discovered that distinct miRNAs are connected with particular subtypes of stroke. For instance, miR-125a-5p and miR-150 have both been linked to cardioembolic strokes and major artery atherosclerosis, respectively. These subtype-specific miRNAs may help with individualized treatment plans by acting as biomarkers for subtype categorization.

Furthermore, there is a link between the magnitude of a stroke and miRNA expression levels. For example, higher levels of miR-424 and miR-320a have been linked to more severe strokes. It may be possible to forecast the severity of a stroke and customize the right treatment approaches by evaluating the expression levels of these miRNAs.

Thus, microRNAs have demonstrated potential as biomarkers for stroke risk assessment. Finding stroke-specific miRNA signatures, comprehending their diagnostic and prognostic value in ischemic strokes, and investigating their associations with stroke subtypes and severity can aid in the development of novel therapeutic targets, more individualized treatment plans, and better stroke management.

### Mechanisms of microRNA-mediated risk stratification

#### Regulation of vascular inflammation by microRNA

Small non-coding RNA molecules called microRNAs (miRNAs) are essential for post-transcriptional gene control. In addition to controlling vascular inflammation, they are engaged in a number of physiological and pathological processes. Vascular inflammation has a significant role in the onset and development of cardiovascular disorders including atherosclerosis (16–18).

A number of miRNAs have been shown to control vascular inflammation. These miRNAs have the ability to target particular genes implicated in inflammatory pathways, which affects how vascular cells respond to inflammation. For instance, miR-155, a key regulator of inflammation, has been demonstrated to enhance vascular inflammation by inhibiting the nuclear factor-kappa B (NF-B) pathway. On the other side, miR-146a targets important components in the toll-like receptor (TLR) signaling pathway and functions as a negative regulator of inflammation (15).

Dysregulation of miRNA expression may promote chronic inflammation and the development of vascular disorders by causing an imbalance in the inflammatory response. In order to reduce vascular inflammation, miRNAs have become viable therapeutic targets as well as indicators for risk assessment.

#### MicroRNA-mediated effects on endothelial dysfunction

Cardiac failure, hypertension, and atherosclerosis are just a few of the several cardiovascular illnesses that frequently exhibit endothelial dysfunction. By controlling vascular tone, inflammation, and thrombosis, the endothelium is essential in preserving vascular homeostasis. Vasodilation is reduced, oxidative stress is elevated, and inflammatory reactions are amplified in endothelial cells that are dysfunctional (18).

The modulation of endothelial function and dysfunction has been linked to miRNAs. They can specifically target genes related to nitric oxide synthesis, endothelial cell adhesion molecules, and endothelial cell survival pathways, among other genes. For instance, it has been demonstrated that miR-155 and miR-221/222 target eNOS, lowering nitric oxide generation and compromising endothelium-dependent vasodilation.

Endothelial dysfunction and the development of cardiovascular illnesses can be attributed to altered expression of certain miRNAs in endothelial cells. Insights into disease causes and prospects for therapeutic treatments may be gained from the discovery and characterization of miRNAs implicated in endothelial dysfunction.

#### Role of microRNA in thrombus formation and resolution

In hemostasis and the response to vascular damage, thrombus development and resolution are closely controlled processes. Deep vein thrombosis, pulmonary embolism, and stroke are thrombotic diseases that can be caused by the dysregulation of these mechanisms (4, 14).

It has been discovered that miRNAs have a role in the control of thrombus development and resolution. They have the ability to affect platelet activation, coagulation, and fibrinolysis, among other elements of thrombus formation. Adenosine diphosphate receptor P2Y12, which is involved in platelet aggregation, has been revealed to be a target of miR-223, which has been proven to decrease platelet activation.

Additionally, miRNAs have a role in the dissolution of thrombi. They have the ability to alter the expression of fibrinolysis-related genes such as tissue-type plasminogen activator (tPA) and plasminogen activator inhibitor-1 (PAI-1). MiR-30c and miR-223 have been demonstrated to control the expression of tPA and PAI-1, respectively, affecting fibrinolysis and thrombus resolution.

The development of novel therapeutic approaches for the prevention and treatment of thrombotic diseases may be facilitated by a better understanding of the function of miRNAs in the generation and resolution of thrombi (14).

### Potential therapeutic applications

#### Targeting microRNA for stroke prevention and treatment

Targeting certain miRNAs that have been found to be dysregulated in stroke patients shows promise for stroke therapy and prevention. It could be able to modify the expression of genes implicated in stroke etiology and possibly lessen the damage caused by stroke by adjusting the amounts of these miRNAs.

Targeting miRNAs for stroke treatment has been investigated using a variety of strategies. Antisense oligonucleotides (ASOs), also known as antagomirs, are one strategy and are created to attach to certain miRNAs and suppress their action. These compounds may be able to offer neuroprotection and enhance stroke outcomes by preventing the negative effects of dysregulated miRNAs (19).

Another strategy is to utilize synthetic miRNAs called miRNA mimics, which may be delivered into cells to raise the levels of a particular miRNA. By promoting neuronal survival and recovery, this strategy seeks to make up for the decreased expression of protective miRNAs in stroke victims.

The area of miRNA-based treatments is still in its infancy, and additional studies are required to fully comprehend the intricate functions that miRNAs play in stroke pathophysiology and to create efficient and secure therapeutic approaches. Clinical studies are being conducted to assess the effectiveness and safety of therapies based on miRNA in stroke patients.

#### Challenges and future directions in microRNA-based therapeutics

While targeting miRNAs for therapeutic purposes shows promise, there are several challenges and future directions that need to be addressed (Table 3).

**Table 3:** Challenges and future directions in microRNAs- based therapeutics (16, 20).

Sr. no	Challenges	Description
1	Delivery	MiRNA-based therapies are difficult to deliver to the target tissues or cells. The delivery method must be effective, secure, and capable of getting to the intended location of action, which in a stroke situation would be the brain. There is ongoing research into creating efficient delivery systems, including those that utilize viral vectors or nanoparticles.
2	Specificity	To prevent off-target impacts and unforeseen consequences, target specificity must be attained. It is crucial to make sure that miRNA-based treatments regulate the targeted miRNA only, with no adverse effects on associated miRNAs or disruption of regular cellular processes.
3	Pharmacokinetics and stability	To achieve the intended effects, miRNA-based treatments must have sufficient stability and half-life in the body. MiRNA mimics or inhibitors can have their chemical structure or chemical backbone altered to increase their stability and improve their pharmacokinetic characteristics.
4	Safety and off-target effects	It is essential to carefully evaluate any potential off-target effects and safety profiles. MiRNA-based therapies have a lot of potential, but they might have unanticipated negative consequences if they interact with other biological functions. In-depth preclinical and clinical research is required to assess the effectiveness and safety of miRNA-based therapies.
5	Biomarker discovery and validation	For patient screening and treatment efficacy monitoring, it is crucial to identify reliable biomarkers linked to particular illnesses and treatment response. The implementation of miRNA-based treatments into clinical practice will be aided by the validation of miRNA biomarkers and the development of reliable diagnostic assays.
6	Combination therapies	MiRNA-based treatments may be more successful when combined with other treatment methods, such as conventional pharmacotherapy or physical therapy, because disorders like stroke are multifactorial in nature. Future studies might examine combinatorial methods and synergistic interactions.

### Future perspectives

Although microRNAs are crucial in ischemic strokes, further study is required to confirm and standardize their signatures. The predictive power of microRNAs for stroke recurrence and long-term outcomes must be determined by longitudinal investigations. Prospective studies can shed light on how the expression of microRNAs varies over time and how that affects the risk of stroke (20, 21).

The precision of risk stratification models can be increased by combining microRNA signatures with other biomarkers, such as imaging characteristics or clinical risk scores. Genomic, transcriptomic, and proteomic data can provide us a more complete knowledge of the pathogenesis of stroke. To gain insight into underlying processes and prospective treatment targets, more research is required to clarify the precise targets and pathways controlled by stroke-associated microRNAs.

Extensive preclinical and clinical investigations are needed for microRNA-based treatments in order to optimize delivery strategies, evaluate off-target effects, and ensure safety and effectiveness. MicroRNAs show significant potential as useful tools for ischemic stroke risk stratification, opening up new opportunities for enhancing stroke therapy and patient outcomes. To fully use the therapeutic potential of microRNAs in ischemic stroke risk assessment and customized treatment, more study is required.

MicroRNAs (miRNAs) offer significant potential in improving the clinical management of ischemic strokes by serving as reliable biomarkers for risk stratification. Their incorporation into clinical workflows could enhance early detection, enable personalized treatment plans, and provide tools for monitoring therapeutic responses. Developing non-invasive diagnostic assays, such as blood-based miRNA panels, could further bridge the gap between laboratory findings and real-world applications, facilitating more precise and proactive patient care.

## Conclusion

With several studies demonstrating their potential for predicting stroke risk, determining the severity of strokes, and predicting clinical outcomes, microRNAs have emerged as promising biomarkers for the risk stratification of ischemic strokes. These tiny non-coding RNAs participate in a number of biological processes, including apoptosis, angiogenesis, and inflammation, all of which are important in the pathophysiology of ischemic strokes. Researchers have discovered distinctive fingerprints connected to various stroke subtypes and risk levels by examining the expression patterns of certain microRNAs. Additionally, microRNAs can be used to predict the severity of a stroke and assist clinicians make treatment decisions, improving patient care and outcomes. Finally, altering the expression levels of certain microRNAs with antagonists or mimics has shown promise in animal models, demonstrating the value of microRNAs as therapeutic targets. This creates new opportunities for targeted medicines and customized therapeutic approach.

## Data Availability

The raw data supporting the conclusions of this article will be made available by the authors, without undue reservation.
